# Clinical outcomes following surgical treatment of peri-implantitis at grafted and non-grafted implant sites: a retrospective analysis

**DOI:** 10.1186/s40729-018-0135-5

**Published:** 2018-08-09

**Authors:** Ausra Ramanauskaite, Kathrin Becker, Gintaras Juodzbalys, Frank Schwarz

**Affiliations:** 1 Department of Oral Surgery, Westdeutsche Kieferklinik, Universitätsklinikum Düsseldorf, D-40225 Düsseldorf, Germany; 2 Clinic of Dental and Oral Pathology, Lithuanian University of Health Sciences, Kaunas, Lithuania; 3 Department of Orthodontics, Westdeutsche Kieferklinik, Universitätsklinikum Düsseldorf, D-40225 Düsseldorf, Germany; 4 Department of Oral and Maxillofacial Surgery, Lithuanian University of Health Sciences, LT-46383 Kaunas, Lithuania; 5 Department of Oral Surgery and Implantology, Carolinum, Johann Wolfgang Goethe-University Frankfurt, D-60596 Frankfurt am Main, Germany

**Keywords:** Peri-implantitis, Diagnosis, Treatment

## Abstract

**Background:**

This retrospective analysis aimed at comparing the clinical outcomes following combined surgical therapy of peri-implantitis at initially grafted and non-grafted (i.e., pristine) implant sites.

**Methods:**

A total of 39 patients exhibiting 57 implants diagnosed with peri-implantitis (i.e., 16 implants at grafted and 41 implants at non-grafted sites) were included. Each subject had received a combined (i.e., implantoplasty and augmentative therapy) surgical treatment procedures at respective implants (grafted sites: 10 patients, 16 implants, non-grafted sites: 29 patients, 41 implants). A chi-squared test (χ^2^) was used to assess whether the initial grafting procedure did affect the treatment outcomes (i.e., disease resolution, bleeding on probing (BOP), probing pocket depths (PD)). The mean follow-up period was 41.9 ± 34.75 months.

**Results:**

At the patient level, disease resolution (i.e., absence of BOP and PD ≥ 6 mm) was obtained in 4/10 (40%) at grafted and in 7/27 (24.1%) at non-grafted implant sites (*p* = 0.579). BOP reductions was found to be 60.64 ± 40.81% at non-grafted and 77.45 ± 30.92% at grafted sites (*p* = 0.778). PD reductions amounted to 2.20 ± 2.22 mm at non-grafted and 1.57 ± 1.54 mm at grafted sites (*p* = 0.969).

**Conclusions:**

The initial bone-grafting procedures at the implant sites did not influence the effectiveness of combined surgical therapy of peri-implantitis.

## Background

Peri-implantitis is caused by a bacterial challenge and characterized by inflammation in the peri-implant soft tissues and a progressive loss of supporting bone [1, 2]. Consequently, its treatment is cause-related and primarily aimed at arresting disease progression [3].

Based on the currently available evidence, non-surgical mechanical debridement alone seems to have a limited efficacy for the management of peri-implantitis [4, 5]. While adjunctive (i.e., local antibiotics, antimicrobial photodynamic therapy) or alternative measures (e.g. air abrasive devices, Er:YAG laser monotherapy) may improve the efficacy of non-surgical therapy, the obtained clinical outcomes appeared to be limited to a period of 6 to 12 months and were particularly compromised at advanced defect sites [4, 5]. In contrast, the efficacy of treatment was commonly improved subsequent to a surgical intervention combining open flap debridement either with adjunctive resective (e.g., apical flap, osteoplasty, implantoplasty (IP)), augmentative (e.g., bone fillers/autografts, guided bone regeneration), or a combination of resective (i.e., IP) and augmentative (refers to as combined therapy) measures [6]. Nevertheless, the reported outcomes following surgical therapy of peri-implantitis varied considerably and appeared to be influenced by a variety of different prognostic factors, such as the configuration of the bony defect [7], the physicochemical properties of the bone filler [8, 9], or the surface characteristics of the affected implants [10, 11].

Previous clinical data provide some evidence that ridge augmentation using either autogenous bone or different bone filler materials may constitute a potential risk indicator for the onset of peri-implant diseases [12, 13]. Consequently, it might be hypothesized that initial bone-grafting procedures at implant site may also influence the effectiveness of peri-implantitis treatment. Therefore, this retrospective analysis aimed at comparing the clinical outcomes following combined surgical treatment of peri-implantitis at initially grafted and non-grafted (i.e., pristine) implant sites.

## Methods

### Study design and participants

For this retrospective analysis, standardized clinical record forms of a total of 39 partially/fully edentulous patients (25 female and 12 male) exhibiting 57 implants were screened. All patients had attended the Department of Oral Surgery, Heinrich Heine University, Düsseldorf, Germany for the treatment of peri-implantitis between 2007 and 2010, and were under regular implant maintenance care. The mean follow-up time was 41.9 ± 34.75 months (range 6 to 126 months). Some patients were also participating in a randomized prospective clinical study, which aimed at investigating the effects of two surface decontamination methods on the clinical outcomes following combined therapy [14].

A data extraction template was generated and used for the anonymous acquisition of demographic study variables/implant site characteristics and baseline as well as follow-up clinical measurements after surgical therapy. The study was in accordance with the Helsinki Declaration, as revised in 2013 and approved by the local ethics committee.

### Patient selection

For patient selection, the following inclusion criteria were defined:

1. Partially or fully edentulous patients rehabilitated with fixed or removable implant-supported prostheses;
2. Presence of at least one screw-type (one or two part) titanium implant diagnosed with peri-implantitis;
3. Respective implants had received a combined surgical peri-implantitis treatment;
4. No implant mobility;
5. Presence of at least 2 mm of keratinized mucosa;
6. Treated chronic periodontitis and proper periodontal maintenance care;
7. A good level of oral hygiene as evidenced by a plaque index (PI) at the implant level < 1;
8. No systemic diseases which could influence the outcome of the therapy (i.e., diabetes (HbA1c < 7), osteoporosis, antiresorptive therapy);
9. No history of malignancy, radiotherapy, chemotherapy, or immunodeficiency within the last 4 years and;
10. Non-smoker or light smoking habits (< 10 cigarettes per day);
11. Complied with at least 6 months of follow-up;
12. Information on the initial bone grafting procedure and protocol at the respective implant site was available.

Patients whose data files lacked information on the bone grafting procedures at the implant site or lacked information on augmentation protocols (i.e., lateral ridge augmentation or sinus floor elevation; one- or two-stage approach), and patients who did not comply with at least 6 months of follow-up were not included in the analysis.

### Case definition

Peri-implantitis was defined as bleeding on probing (BOP) with or without suppuration (Supp) in addition to changes in the radiographic bone level. Interproximal bone level changes were estimated on intraoral radiographs. In the absence of available baseline radiographs taken at prosthesis installation, “a threshold vertical distance of 2 mm from the expected marginal bone level” was used to assess bone loss [3].

### Initial grafting procedures

The identified patients with a history of grafting had received the following treatment protocols:

#### Lateral ridge augmentation

- Simultaneous grafting (one stage) of dehiscence-type defects, employing a particulated bone substitute, and collagen membrane (2 patients; 3 implants)
- Grafting and staged implant placement at 6 months (two stage) employing a particulated bone substitute and collagen membrane (3 patients; 3 implants)

#### Sinus floor elevation

- External grafting (lateral window) employing a particulated bone substitute and collagen membrane and implant placement (2 patients; 3 implants)
- External grafting (lateral window) employing a particulated bone substitute and collagen membrane and staged implant placement at 6 months (4 patients; 7 implants)

To be included, the radiographic bone loss at baseline (i.e., prior to treatment) in respective patients had to extend to the formerly grafted area.

### Treatment procedures

**Fig. 1:**
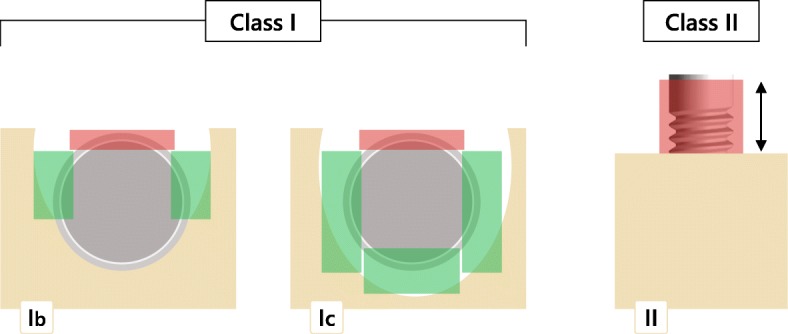
Combined surgical therapy of peri-implantitis at respective defect sites: class I: intrabony component showing either a buccal dehiscency with a semicircular component (Ib) or a buccal dehiscency with a cicumferential component (Ic). Class II: supracrestal component. The red rectangles indicate the surface areas undergoing an implantoplasty, while the green areas indicate the defect areas undergoing augmentative therapy

After an initial course of non-surgical therapy, each subject had received a combined (i.e., implantoplasty + augmentative therapy) surgical treatment procedure [14] at respective implant sites (Fig. 1). This procedure included open flap debridement and a meticulous granulation tissue removal using conventional plastic curets (Straumann Dental Implant System; Institut Straumann AG, Basel, Switzerland) and an implantoplasty at both buccally (i.e., Classes Ib and Ic) and supracrestally (i.e., class II) (Fig. 1) exposed implant surfaces. This was accomplished using diamond burs (ZR Diamonds; Gebr. Brasseler GmbH & Co. KG, Lemgo, Germany) and Arkansas stones under copious irrigation with sterile saline. The remaining unmodified implant surfaces at the respective intrabony defect areas (i.e., classes Ib, Ic, and Ie) were decontaminated using either an Er:YAG laser device (energy density of 11.4 J/cm2, 10 Hz) (elexxion delos; elexxion AG, Radolfzell, Germany) or debrided using plastic curetes and cotton pellets soaked in sterile saline (Straumann Dental Implant System). Respective intrabony defect compartments were homogeneously filled using NBM (BioOss spongiosa granules, particle size 0.25–1 mm; Geistlich, Wolhusen, Switzerland) and were covered with CM (BioGide; Geistlich). Transmucosal healing was supported by a peri- and postoperative antibiotic medication for 5 days.

### Clinical examination

For all patients, the following clinical parameters were available: BOP (as measured within 60 s after probing) and PD (as measured in millimeters from the mucosal margin to the bottom of the probeable pocket). BOP and PD were assessed at six aspects around the implant: mesio-buccal, mid-buccal, disto-buccal, mesio-oral, mid-oral, and disto-oral. Maximum PD values (max PD) and mean BOP scores were evaluated before the surgical intervention and at the final follow-up.

The primary outcome variable was disease resolution (i.e., the composite outcome of the absence of BOP and probing pocket depths (PD ≥ 6 mm). Reduction of mean BOP and maximum PD values were defined as secondary outcome variables.

### Data analysis

Commercially available and open source software programs (SPSS Statistics 23.0: IBM Corp., Ehningen, Germany and R Development Core Team) were used. Mean values, standard deviations (SD), medians, minimums, and maximums were calculated for mean BOP and maximum PD scores.

The analyses were performed at both patient and implant levels. Prior to this analysis, clinical parameters were pooled according to the grafting procedure (grafted or non-grafted), considering the patient as statistical unit. The differences in the baseline maximum PD values between the grafted and non-grafted implant sites were assessed using Wilcoxon rank-sum test. To evaluate disease resolution, changes in mean BOP and maximum PD between the groups (i.e., non-grafted vs. grafted) chi-square test (χ^2^) were applied. For the evaluation of disease resolution, if patients exhibited multiple implants with different treatment outcomes, they were assigned to a group according to the worst one. Based on the sample size calculation, for a large effect size (*w* = 0.5, df = 1, alpha = 0.05, power = 0.8), a minimum of 32 patients were needed [15].

The results were considered statistically significant at *p* < 0.05.

## Results

The present analysis was based on 39 patients diagnosed with peri-implantitis in 57 implants. The patients were divided into 2 groups according to the grafting of the site: non-grafted implant sites (29 patients/41 implants) and grafted implant sites (10 patients/16 implants).

The characteristics of the implant sites are presented in Table 1. In total, 26 implants (45.6%) were located in the maxilla and the remaining 31 (54.4%) were located in the mandible. Out of these implants, 15 (26%) were located in the anterior (incisor and canine area) and 42 (74%) were located in the posterior regions (premolars and molars). All the studied implants (100%) presented with BOP, and only 1 implant had BOP in fewer than 6 sites.

**Table 1:** Implant site characteristics

	Non-grafted sites	Grafted sites
Implant number	41	16
Maxilla/mandible	19/22	7/9
Anterior/posterior	10/31	5/11
Baseline max PD* values (mm)		
Mean	6.77	6.25
SD	1.61	0.45
Median	6.0	6.0
Minimum	3.0	6.0
Maximum	10.0	7.0

*No significant difference in baseline maximum PD values between the groups was found (Wilcoxon *p* = 0.353)

**Fig. 2:**
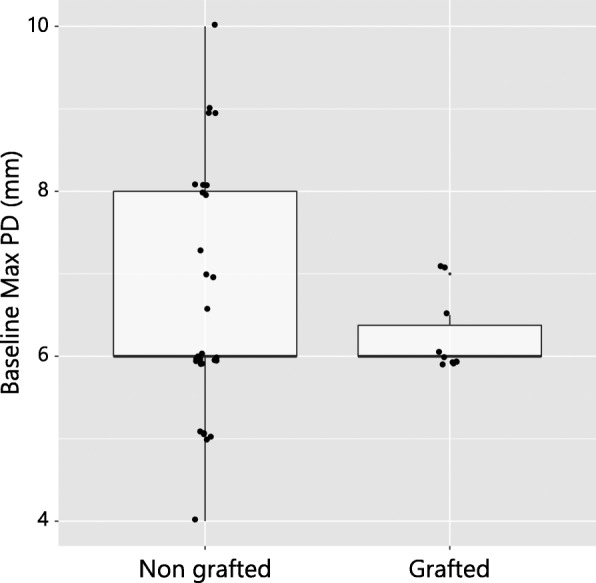
Box plot depicting no significant differences of the baseline maximum PD values between the grafted and non-grafted patient groups (*p* = 0.353)

At the baseline, the maximum PD values at the grafted and non-grafted sites 6.25 ± 0.45 mm and 6.77 ± 1.61 mm, respectively (Table 1, Fig. 2). The Wilcoxon rank-sum test did not show a significant difference in the baseline maximum PD values between the groups (*p* = 0.353) (Table 1).

### Disease resolution

**Fig. 3:**
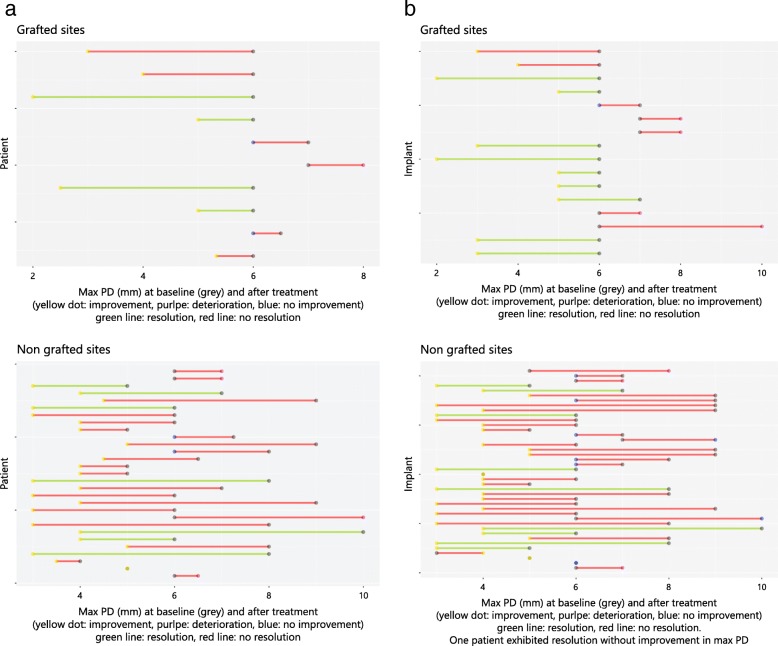
Dumbbell—dot plots illustrating (**a**) disease resolution and no resolution at non-grafted and grafted sites at the patient level analysis with no significant difference between the two groups (*p* = 0.579). Patents with improvement, deterioration, and no improvement following the treatment based on the max PD changes are depicted; (**b**) disease resolution and no resolution at the implants in non-grafted and grafted sites. Significantly higher disease resolution in the grafted implant group (*p* = 0.048)

In general, disease resolution (i.e., the absence of BOP and PD ≥ 6 mm) was achieved in 19 out of 57 (33%) implants and in 11 out of 39 (28%) patients. At the patient level, disease resolution was obtained in 4 out of 10 patients (40%) at grafted sites and in 7 out of 29 patients (24.1%) at non-grafted sites. The chi-square test (χ2) demonstrated no significant difference between the two patient groups (*p* = 0.579, df = 1, χ2 = 0.307) (Table 2, Fig. 3a). However, the results of the implant-level analysis revealed a significant difference between the two groups, indicating a higher disease resolution at grafted implant sites (9/16 (56%) compared to non-grafted sites (10/41 (25%)) (*p* = 0.048, df = 1, χ2 = 3.921) (Fig. 3b).

**Table 2:** Disease resolution between the non-grafted and grafted implant sites

	Non-grafted sites	Grafted sites	Total
Patient level	7/29 (24.1%)	4/10 (40%)	11/39 (28%)
Implant level	10/41 (25%)	9/16 (56.3%)	19/57 (33%)

No significant difference between the groups was found at the patient level (*p* = 0.579, chi-square test). Significantly higher disese resolution was achieved in grafted implant sites at the implant level (*p* = 0.048, chi-square test)

### BOP reduction

Mean and median BOP reduction values (%) for the 2 groups are presented in Table 3. At the patient level, mean BOP reduction amounted to 77.45% (minimum 0%; maximum 100%) and 60.64% (minimum 0%; maximum 100%) at grafted and non-grafted sites, respectively.

**Table 3:** Reduction of mean BOP (%)

Group	Patient level					Implant level				
	mean	SD	median	min.	max.	mean	SD	median	min.	max.
Non-grafted sites	60.64	40.81	67.0	0	100	54.88	43.65	67.0	0	100
Grafted sites	77.45	30.92	87.25	0	100	74.96	38.95	100	0	100

The differences between the groups did not yield a significant difference (patient level *p* = 0.778, implant level *p* = 0.515, chi-square test)

**Fig. 4:**
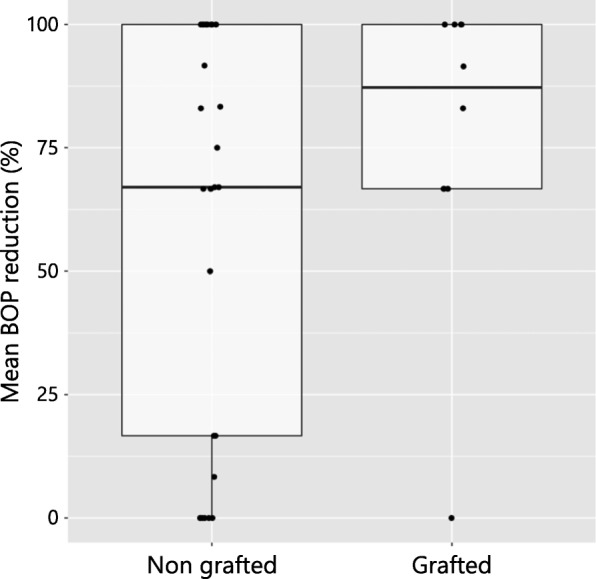
Box plot presenting mean BOP reduction between the two patient groups (grafted and non-grafted) with no significant difference (*p* = 0.778)

At the implant level, BOP reduction was noted to be 74.96% (minimum 0%; maximum 100%) at grafted implant sites and 54.88% (minimum 0%; maximum 100%) at non-grafted implant sites. According to the results of the chi-square test, the mean BOP reduction did not differ significantly between the groups at either the patient (*p* = 0.778, df = 1, χ2 = 0.079) or the implant (*p* = 0.515, df = 1, χ2 = 0.422) level (Fig. 4).

### PD changes

**Fig. 5:**
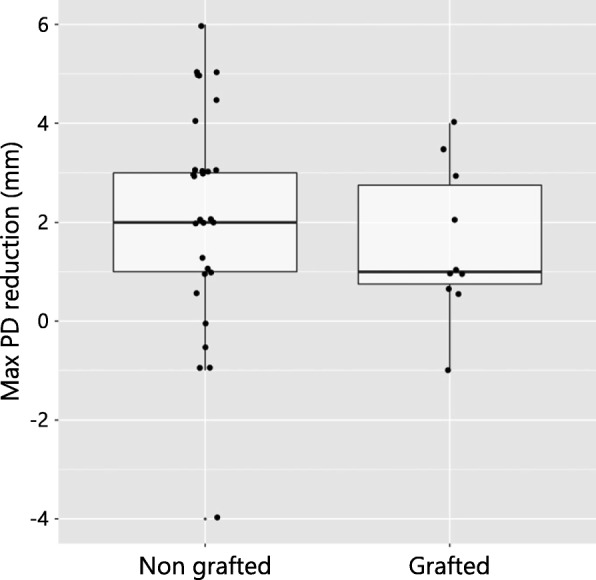
Box plot illustrating maximum PD reduction between the grafted and non-grafted patient groups that did not reach a significant difference (*p* = 0.968)

The reduction in the maximum PD (mm) values between the 2 groups is presented in Table 4. At the patient level, reduction in the maximum PD values amounted to 1.57 mm (minimum − 1.0 mm; maximum 4 mm) and 2.20 mm (minimum: − 4.0 mm; maximum 6.0 mm) at grafted and non-grafted sites, respectively. At the implant level, the corresponding reduction in the maximum PD values were calculated to be 1.31 mm (minimum − 4.0 mm; maximum 4.0 mm) at grafted implant sites and 2.10 mm (minimum − 4.0 mm; maximum 6.0 mm) at non-grafted implant sites. For the between-group comparison, no significant difference could be detected between the groups at both the patient (*p* = 0.968, df = 1, χ2 = 0.002, chi-square test) and implant (*p* = 1, df = 1, χ2 = 0.00026, chi-square test) level (Fig. 5).

**Table 4:** Reduction of maximum PD (mm)

Group	Patient level					Implant level				
	Mean	SD	Median	Min.	Max.	Mean	SD	Median	Min.	Max.
Non-grafted sites	2.20	2.22	2.0	−4.0	6.0	2.10	2.35	2.0	−4.0	6.0
Grafted sites	1.57	1.54	1.0	−1.0	4	1.31	2.18	1.5	−4.0	4.0

No significant difference between the groups was observed (patient level *p* = 0.968, implant level *p* = 1, chi-square test)

## Discussion

According to the eighth European Workshop of Periodontology (EFP), evaluation of the effectiveness of different peri-implantitis therapies should be based on a composite outcome of disease resolution, including resolutions of mucosal inflammation, reductions in probing pocket depths, and no further bone loss [3].

The current retrospective clinical investigation evaluated treatment outcomes following combined surgical therapy for peri-implantitis at formerly grafted and non-grafted implant sites. A composite outcome of disease resolution as the absence of BOP and PD ≥ 6 mm was considered. Accordingly, peri-implantitis resolution was achieved in 33% of the treated implants, corresponding to 28% of the patients. Although no significant difference regarding grafting of the implant site was detected at the patient level (grafted sites 4/10 (40%), non-grafted sites 7/29 (24.1%), *p* = 0.579), implant-level analysis pointed to a higher disease resolution at the grafted implant sites (9/16 (56%) at grafted sites, 10/41 (25%) at non-grafted sites, *p* = 0.048).

The disease resolution noted in the present analysis is in line with the data reported in previous clinical studies. In particular, the treatment success (defined as absence of BOP) following combined surgical therapy was obtained in 60% (9/15) of the patients in the 7 years of clinical investigation [16]. Additionally, according to the results of the studies reporting on the composite treatment outcomes following surgical regenerative peri-implantitis therapy, treatment success was achieved in 35% (9/26) (treatment success defined as PD < 5 mm, absence of BOP/suppuration, no further bone loss) [17] to 51.1% (23/45) of the implants (treatment success defined as evidence of ≥ 25% bone fill, PD < 5 mm, BOP score ≤ 1) at 5 and 7 years of follow-up, respectively [18]. However, in this context, it should be realized that these studies used different criteria to define treatment success; hence, clinical outcomes cannot be compared directly.

In the present study, mean BOP reduction ranged from 60.64 to 77.45% at the patient level and from 54.88 to 74.96% at the implant level, with no significant difference between the grafted and non-grafted implants sites. Slightly higher mean BOP reduction values, ranging from 75.5 to 90%, were indicated in the long-term (7 years) clinical investigations following regenerative surgical therapy of peri-implantitis [16, 17]. It is interesting to note that BOP reduction was found to be significantly influenced by the implant-surface characteristics [17]. This observation is in agreement with the data presented in a 3-year randomized controlled clinical trial, where superior treatment outcomes were noted for implants with non-modified surface implants compared to modified surfaces [11].

The further evaluation of maximum PD reduction did not indicate a significant difference between the two groups (i.e., grafted vs. non-grafted), with the range of 1.57 to 2.20 mm at the patient level analysis and 1.31 to 2.10 mm at the implant level. These results are in concurrence with data from the previous studies, where mean PD reduction amounted from 0.74 to 2.55 mm [16], up to 3 [18], and > 4 mm [17] following surgical regenerative peri-implantitis therapy. To the authors’ best knowledge, this is the first clinical study to evaluate peri-implantitis treatment outcomes at formerly grafted and non-grafted implant sites. Therefore, the results (e.g., disease resolution, changes in mean BOP, and maximum PD) cannot be compared to those from previous studies.

A recent systematic review and meta-analysis indicated lateral bone grafting procedures (both simultaneous with implant placement and staged) to be associated with peri-implant tissue stability [19]. Particularly, the results, which were based on eight clinical investigations, addressed that different surgical interventions (i.e., GBR and autogenous, allogeneic, or xenogeneic bone blocks) resulted in low and similar BOP values, along with comparable PD, marginal bone and plaque levels at both short-term (1–3 years) and long-term (> 3 years) follow-ups [19]. Moreover, the occurrence of peri-implant diseases or a progressive marginal bone loss was reported to be low and also comparable between different lateral hard tissue grafting protocols [20]. However, the data on whether former bone grafting procedures influence peri-implantitis treatment outcomes has not been reported in the literature.

Based on the findings of the current investigation, combined surgical treatment was associated with clinically important reduction in BOP and PD values and was not influenced by the presence or absence of the grafting procedure at the implant site. When interpreting these results, the relatively small number of patients in the group with grafted implant sites should be taken into consideration. Additionally, it should be noted that two different decontamination protocols (i.e., Er: YAG laser or debriding with plastic curettes and cotton pellets soaked in sterile saline) were applied. Nevertheless, according to the findings from the randomized clinical trial, the method used to decontaminate the implant surface had no impact on the clinical outcomes of the combined surgical therapy of peri-implantitis [16].

## Conclusions

Within the limitations of the current study, it was concluded that the effectiveness of combined surgical therapy of peri-implantitis was comparable at both grafted and non-grafted implant sites and was not influenced by the initial bone-grafting procedures.
